# Comparison of Intraocular Pressure, Blood Pressure, Ocular Perfusion Pressure and Blood Flow Fluctuations During Dorzolamide *Versus* Timolol Add-On Therapy in Prostaglandin Analogue Treated Glaucoma Subjects

**DOI:** 10.3390/ph5030325

**Published:** 2012-03-21

**Authors:** Ingrida Januleviciene, Lina Siaudvytyte, Vaida Diliene, Ruta Barsauskaite, Daiva Paulaviciute-Baikstiene, Brent Siesky, Alon Harris

**Affiliations:** 1 Department of Ophthalmology, Hospital of Lithuanian University of Health Sciences Kaunas Clinics, Eye Clinic of Kaunas University of Medicine, Eiveniu Str. 2, Kaunas 50009, Lithuania; 2 Glaucoma Research and Diagnostic Center, Indiana University School of Medicine, Indianapolis, IN 46202, USA

**Keywords:** retrobulbar blood flow, intraocular pressure, ocular perfussion pressure, color dopler imaging, open-angle glaucoma

## Abstract

Objective: To compare the effects of dorzolamide and timolol add-on therapy in open-angle glaucoma (OAG) patients previously treated with prostaglandin analogue (Pg), by evaluating fluctuations in the intraocular (IOP), blood (BP), ocular perfusion pressures (OPP) and retrobulbar blood flow (RBF) parameters. Methods: 35 OAG patients (35 eyes), 31 women (88.6%) age 63.3 (8.9) years were evaluated in a 3 month randomized, cross-over, single-masked study. During the experiments BP, heart rate, IOP and OPP were assessed 4 times per day (8–12–16–20 h). RBF was measured twice per day (8–20 h) using Color Doppler imaging in the ophthalmic (OA), central retinal (CRA), nasal (nSPCA) and temporal (tSPCA) posterior ciliary arteries. In each vessel, peak systolic velocity (PSV) and end-diastolic velocity (EDV) were assessed and vascular resistance (RI) calculated. Results: Both add-on therapies lowered IOP in a statistically significant manner from 15.7 ± 2.4 mmHg at latanoprost baseline to 14.9 ± 2.2 mmHg using dorzolamide (*p* < 0.001) and 14.2 ± 1.9 mmHg using timolol (*p* < 0.001). The IOP lowering effect was statistically significant at 20 h, favoring timolol as compared to dorzolamide (1.4 ± 2.4 *vs*. 0.2 ± 2.1 mmHg), (*p* < 0.05). Dorzolamide add-on therapy showed smaller IOP (2.0 ± 1.4), SPP (13.3 ± 7.9), systolic BP (13.5 ± 8.7) and diastolic BP (8.4 ± 5.4) fluctuations as compared to both latanoprost baseline or timolol add-on therapies. Higher difference between morning and evening BP was correlated to decreased evening CRA EDV in the timolol group (c = −0.41; *p* = 0.01). With increased MAP in the morning or evening hours, we found increased evening OA RI in timolol add-on group (c = 0.400, *p* = 0.02; c = 0.513, *p* = 0.002 accordingly). Higher MAP fluctuations were related to impaired RBF parameters during evening hours-decreased CRA EDV (c = −0.408; *p* = 0.01), increased CRA RI (c = 0.576; *p* < 0.001) and tSPCA RI (c = 0.356; *p* = 0.04) in the dorzolamide group and increased nSPCA RI (c = 0.351; *p* = 0.04) in the timolol add-on group. OPP fluctuations correlated with increased nSPCA RI (c = 0.453; *p* = 0.006) in the timolol group. OPP fluctuations were not related to IOP fluctuations in both add-on therapies (*p* < 0.05). Conclusions: Both dorzolamide and timolol add-on therapies lowered IOP in a statistically significant fashion dorzolamide add-on therapy showed lower fluctuations in IOP, SPP and BP. Higher variability of daytime OPP led to impaired RBF parameters in the evening.

## 1. Introduction

Several large, multicenter, randomized clinical trials have shown that in many cases glaucoma continues to progress, despite maintaining target intraocular pressure (IOP) [1,2] The contribution of vascular deficits in glaucomatous optic neuropathy (GON) is based upon a recent increase in clinical data on vascular dysregulation involved in glaucoma pathology and was reflected by the World Glaucoma Association consensus on ocular blood flow (OBF) in glaucoma [3].

Non-IOP factors such as lower systolic ocular perfusion pressure (OPP), reduced OBF, cardiovascular disease, low systolic blood pressure (BP) have been identified as risk factors for primary GON. The Beaver Dam study reported a positive correlation between systolic BP and IOP [4]. Evidence suggest that subjects with glaucoma fail to adapt to changes either in IOP or BP that cause fluctuations in OPP, ultimately resulting in unstable blood flow to the retina and optic nerve head [5,6]. The day-night variations of IOP have been previously analyzed in healthy subjects and in primary glaucoma patients [7]. Asrani and colleagues pointed the risk associated with diurnal IOP variations in patients with open-angle glaucoma and showed the importance of controlling IOP throughout the day [8]. Nocturnal overdipping of BP may lead to optic nerve ischemia and be an important IOP independent risk factor [3].

Our current knowledge of the vascular anatomy and physiology has increased over the past decade allowing us to identify the regional vascular beds involved in glaucoma. Imaging technologies allow non-invasive assessment of the ocular circulation. 

It is still debated if changes in OBF are a consequence of IOP changes or represent a primary physiological response. Quaranta *et al*. analyzed 24-hour IOP and BP changes with different medications, but ocular blood flow parameters were not analyzed [9]. Some studies reported that dorzolamide besides pressure-lowering action, can have a vasodilatative action due to the induced acidosis in local tissues. Differently than dorzolamide, timolol may cause vasoconstriction by blocking β-2 receptors [10] and is more effective on lowering IOP [11]. Ocular hypotensive medications may have the potential to improve OBF in the ocular tissues, but it is difficult to discriminate between the effects of the lowered IOP and those of the improved circulation [3]. Although changes in ocular blood flow might be the consequence of IOP variations, they can also be a primary physiological event [12]. It is therefore important not only to identify ocular blood flow deficits in GON, but also document the influence of glaucoma treatment on the ocular circulation. 

The main idea of our study was to analyse whether dorzolamide add-on therapy is statistically different from timolol treatment, whether fluctuations in IOP, OPP, BP are linked to impaired ocular blood flow in OAG patients and whether fluctuations of OPP are related with the IOP fluctuations.

## 2. Objectives

To compare the dorzolamide and timolol add-on effect in patients already treated with the prostaglandin analogue (Pg) latanoprost, evaluating IOP, BP, OPP and retrobulbar blood flow fluctuation parameters.

## 3. Materials and Methods

Forty OAG patients were recruited into the randomized, cross-over, single-centre masked 3 month study. All study procedures were carried out according to the Declaration of Helsinki, and the study protocol was approved by the Kaunas Universisty of Medicine Review Board. For the objectivity of data the Reading Center Glaucoma Research and Diagnostic Center, Indiana University School of Medicine (USA) served as the double blind reading center site and analyzed all blood flow and perfusion data along with study coordination. 

Study sample calculation was based on previous studies [13] and test/retest variability of the color Doppler and transcranial Doppler measurement of retrobulbar flow velocities showing that a sample size of 20 individuals per treatment arm will provide 80% power to detect a 8 cm/s increase in OA PSV and 4 cm/s in OA EDV also 5 cm/s increase in CRA PSV and 4 cm/s increase in CRA EDV between the control and the glaucoma groups.

Best corrected visual acuity (LogMar test), perimetry (24-2 Swedish Interactive Threshold Algorithm standard program on the Humphrey Field Analyser (Carl Zeiss Meditec Inc., Dublin, CA, USA) tests were carried out at the begining and at the end of study period. Reliable visual field test defined as a false positive error of less than 15%, a false negative error of less than 15% and a fixation loss less than 20%. Mean deviation (MD) and pattern standard deviation (PSD) were used for visual field data analysis.

BP, heart rate, IOP, ocular perfusion pressure (OPP) calculated as 2/3 of mean arterial blood pressure minus IOP; diastolic perfusion pressure (DPP) calculated as diastolic blood pressure minus IOP; systolic perfusion pressure (SPP) calculated as systolic blood pressure minus IOP were analysed 4 times per day (8–12–16–20 h).

Color Doppler imaging (CDI; Accuvix, Seoul, Korea) for measurements of blood flow in the ophthalmic (OA), central retinal (CRA), nasal (nSPCA) and temporal (tSPCA) short posterior ciliary arteries was carried out twice daily at 8 and 20 h. In each vessel, peak systolic velocity (PSV) and end diastolic velocity (EDV) were determined, Pourcelot’s resistive index was calculated (RI = (PSV-EDV)/PSV). Both eyes were examined, the study eye was chosen randomly using a randomization table. Patients were instructed to avoid caffeine intake, smoking, and exercise for 2 h prior to study visit.

At the time of enrollment all subjects were receiving binocular Pg analogue (Xalatan, Pfizer, Inc.) monotherapy and rated as subcompensated IOP by refering ophthalmologist. Before the study glaucoma was defined by elevated IOP > 21 mmHg, optic nerve disc cupping and retinal nerve fibber loss charicteristic of glaucoma, presence of visual field defects in perimetry charicteristic of glaucoma, open angles on gonioscopy. After baseline latanoprost measurements, patients were randomly assigned to add-on treatment group A or B (20 patients in each group): group A receiving topical dorzolamide 2% (Trusopt^®^, Merck & Co. Inc.) add-on b.i.d. (morning and bedtime, every 12 h) and group B receiving topical timolol 0.5% (Oftan Timolol, Santen OY) b.i.d. A randomization table was used to randomize eligible patients. Examinations after add-on therapy were carried out after 1 month of treatment. Patients were washed out 4 weeks, receiving only Pg analogue (Latalux, Sanitas, AB) monotherapy and a second set of baseline II parameters were obtained. Patients previously having received dorzolamide were signed for timolol and *vice versa*. After 4 weeks of patient masked treatment the second set of add-on treatments was evaluated.

## 4. Statistical Analysis

The statistical analysis was performed using SPSS 17.0 for Windows. Descriptive statistics presented as number and percentage for categorical variables, and mean ± standard deviation (SD) for continuous variables. Mann-Whitney’s nonparametric test was used when the assumption of the data normality was rejected. The two way ANOVA with the Bonferroni adjustment was performed calculating exact probabilities and considering comparison significant if the p-value was less than 0.05 Changes in individual parameters were examined by paired Student’s t-test. Fluctuations of IOP, BP, OPP and ocular blood flow were modeled using a nonlinear least square dual harmonic regression procedure, studying the mean value, the highest and lowest value and the amplitude of each rhythm. 

Based on statistical mediana patients were divided into two groups for comparison: younger than 65 and 65 years and older; patients with period of illness ≥ and < than 5 years; patients using ≥ and < than 2 systemic medications.

Association between categorical variables or abnormally distributed continuous variables was assessed by Spearman’s correlation. There was no statistically significant diffference between baseline 1 and baseline 2 measurements, therefore basaline 1 was used for all statistical anglysis. The level of significance *p* < 0.05 was considered significant.

## 5. Results

Forty OAG patients were recruited, but five patients (12.5%) withdrew from the study due to difficulties attending all study visits. Data from the remaining 35 OAG patients (88.6% women) who finished all study procedures were included for analysis. Patient characteristics are provided in Table 1. Mean age of the study population was 63.3 ± 8.9 years, mean duration of glaucoma therapy was 6.1 ± 6.3 years. Best corrected visual acuity logMar test was 0.25 ± 0.2 and no statistically significant differences were noted during other study visits.

**Table 1:** Patient characteristics.

	Mean (SD)	Number (percentage)
Age [years]	63.3 (8.9)	
Men (N (%))		4 (11.4%)
Women (N (%))		31 (88.6%)
Glaucoma treatment [years]	6.1 (6.3)	
Patients receiving systemic medications:	2.3 (1.9)	
Beta blockers (N (%))		12 (34.3%)
ACE inhibitors (N (%))		7 (20.0%)
Angiotensin II inhibitors (N (%))		6 (17.1%)
Diuretics (N (%))		6 (17.1%)
Other drugs (N (%))		18 (51.4%)
		
Systolic BP-baseline [mmHg]	139.5 (15.0)	
Diastolic BP-baseline [mmHg]	82.8 (7.1)	
Pulse rate-baseline	69.3 (13.8)	
		
Systolic BP-Dorzolamide add-on [mmHg]	136.1 (14.0)	
Diastolic BP-Dorzolamide add-on [mmHg]	81.2 (8.0)	
Pulse rate-Dorzolamide add-on	71.8 (9.0)	
		
Systolic BP-Timolol add-on [mmHg]	137.6 (14.0)	
Diastolic BP-Timolol add-on [mmHg]	80.7 (8.7)	
Pulse rate-Timolol add-on	68.2 (13.7)	

**Figure 1:**
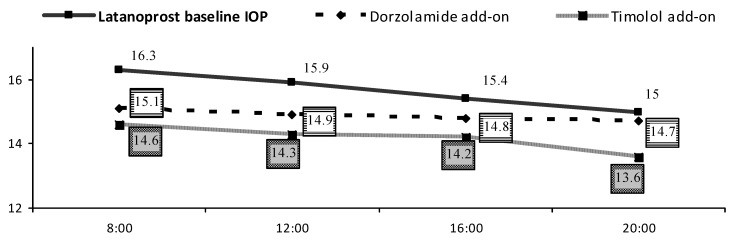
IOP diurnal fluctuations [mmHg].

IOP decreased statistically significantly during both add-on therapies (Figure 1). During latanoprost treatment baseline mean IOP was 15.7 ± 2.4 mmHg and decreased to 14.9 ± 2.2 mmHg using dorzolamide (*p* < 0.001) and to 14.2 ± 1.9 mmHg using timolol (*p* < 0.001). At 20 h the IOP lowering effect was statistically significant, favoring timolol as compared to dorzolamide (1.4 ± 2.4 *vs*. 0.2 ± 2.1 mmHg (*p* < 0.05; t test) (Table 2). Analysing IOP fluctuations during all study visits, IOP was >18 mmHg during 46 (8.2%) visits: 21 and 12 (15% and 8.6%) during latanoprost first and second baseline, 10 (7.1%) during dorzolamide add-on and 3 (2.1%) with timolol add-on. 153 (27.3%) study visits showed IOP < 14 mmHg: 26 and 31 (18.6% and 21%) during latanoprost first and second baseline, 40 (28.6%) with dorzolamide add-on and 56 (40%) adding timolol.

Mean RBF velocities and resistive indices in different treatment groups are shown in Table 3. During timolol add-on therapy evening OA RI increased from baseline 0.77 to 0.79, morning CRA RI increased from 0.69 to 0.71, but differences were not statistically significant (*p* > 0.05; t test).

**Table 2:** Changes in IOP during add-on therapies.

IOP [mmHg]	Mean (SD)			∆		
	Baseline	Dorzolamide	Timolol	Dorzolamide change from baseline		Timolol change from baseline
	15.7 ± 2.4	14.9 (2.2) *	14.2 (1.9) *	8 h	1.2 (2.1)	1.6 (2.1)
				12 h	0.9 (2.6)	1.6 (2.3)
				16 h	0.6 (1.7)	1.2 (1.7)
				20 h	0.2 (2.1)	1.4 (2.4) **

* Paired Samples t test. Significance level *p* < 0.05 (add-on therapy changes from latanoprost baseline). ** Student t test. Significance level *p* < 0.05 (changes between dorzolamide and timolol).

**Table 3:** Color Doppler imaging parameters during different therapies.

	Latanoprost baseline		Dorzolamide add-on		Timolol add-on	
Doppler parameters	Morning Mean (SD)	Evening Mean (SD)	Morning Mean (SD)	Evening Mean (SD)	Morning Mean (SD)	Evening Mean (SD)
OA PSV [cm/s]	33.5 (6.7)	33.6 (2.6)	34.8 (7.7)	35.1 (7.1)	33.3 (6.9)	33.5 (5.8)
OA EDV [cm/s]	7.3 (2.6)	7.5 (2.6)	8.0 (3.2)	7.7 (3.6)	7.4 (2.8)	7.0 (2.4)
						
CRA PSV [cm/s]	11.7 (1.4)	11.8 (1.8)	11.4 (1.9)	12.0 (2.0)	11.6 (2.1)	11.4 (1.9)
CRA EDV [cm/s]	3.5 (0.7)	3.6 (0.7)	3.6 (1.1)	3.5 (1.0)	3.2 (0.9)	3.5 (1.0)
CRA RI	0.69 (0.07)	0.69 (0.07)	0.68 (0.08)	0.70 (0.07)	0.71 (0.08)	0.69 (0.08)
						
nSPCA PSV [cm/s]	8.4 (1.1)	8.4 (1.2)	8.7 (2.0)	8.7 (1.6)	8.5 (1.6)	8.8 (2.0)
nSPCA EDV [cm/s]	3.5 (0.6)	3.5 (0.6)	3.6 (0.9)	3.8 (0.8)	3.7 (1.0)	3.7 (0.9)
nSPCA RI	0.58 (0.07)	0.58 (0.06)	0.58 (0.09)	0.56 (0.09)	0.57 (0.08)	0.58 (0.09)
						
tSPCA PSV [cm/s]	8.2 (1.4)	8.3 (1.3)	8.0 (1.6)	8.4 (1.4)	8.1 (1.7)	8.2 (1.8)
tSPCA EDV [cm/s]	3.4 (0.6)	3.6 (0.6)	3.5 (0.7)	3.5 (0.6)	3.4 (0.6)	3.4 (0.8)
tSPCA RI	0.58 (0.08)	0.56 (0.08)	0.54 (0.11)	0.58 (0.08)	0.56 (0.09)	0.57 (0.11)

OA–ophthalmic artery; CRA–central retinal artery; nSPCA–nasal short posterior ciliary artery; tSPCA–temporal short posterior ciliary artery; PSV–peak systolic velocity; EDV–end diastolic velocity; RI–resistive index.

Changes in diurnal CDI parameters between different patient groups are shown in Table 4. There was a statistically significant difference (*p* < 0.05) in RBF parameters between patients younger than 65 (N = 17) and older than 65 (N = 18).

**Table 4:** Changes of diurnal CDI parameters in different groups.

Patient groups	CDI parameter	Latanoprost baseline Mean (SD)	Dorzolamide add-on Mean (SD)	Timolol add-on Mean (SD)
Age <65 years	OA RI	0.79 (0.05)	0.76 (0.09)	0.77 (0.07)
	CRA RI	0.68 (0.07)	0.67 (0.07)	0.69 (0.07)
	tSPCA RI	0.57 (0.07)	0.56 (0.09)	0.55 (0.08)
	nSPCA RI	0.56 (0.08)	0.55 (0.09)	0.56 (0.09)
				
Age ≥65 years	OA RI	0.78 (0.04)	0.79 (0.05)	0.80 (0.04)
	CRA RI	0.72 (0.04)	0.73 (0.06)	0.71 (0.06)
	tSPCA RI	0.59 (0.05)	0.59 (0.05)	0.61 (0.06)
	nSPCA RI	0.58 (0.06)	0.57 (0.08)	0.58 (0.09)
				
Glaucoma treatment <5 years	OA RI CRA RI	0.77 (0.04) 0.67 (0.06)	0.76 (0.07) 0.66 (0.06)	0.77 (0.07) 0.68 (0.07)
	tSPCA RI	0.56 (0.05)	0.54 (0.07)	0.56 (0.08)
	nSPCA RI	0.55 (0.05)	0.52 (0.07)	0.55 (0.08)
				
Glaucoma treatment ≥5 years	OA RI	0.79 (0.05)	0.80 (0.07)	0.81 (0.05)
	CRA			
	CRA RI	0.73 (0.04)	0.73 (0.05)	0.73 (0.04)
	tSPCA RI	0.60 (0.06)	0.60 (0.06)	0.59 (0.07)
	nSPCA RI	0.59 (0.08)	0.60 (0.08)	0.59 (0.09)
			
Taken systemic medication <2	OA RI	0.78 (0.05)	0.76 (0.08)	0.78 (0.07)
	CRA RI	0.69 (0.06)	0.68 (0.06)	0.69 (0.06)
	tSPCA RI	0.57 (0.05)	0.56 (0.06)	0.58 (0.06)
	nSPCA RI	0.56 (0.06)	0.56 (0.08)	0.57 (0.09)
				
Taken systemic medication ≥2	OA RI	0.79 (0.04)	0.79 (0.06)	0.79 (0.06)
	CRA RI	0.71 (0.06)	0.71 (0.07)	0.72 (0.07)
	tSPCA RI	0.59 (0.06)	0.58 (0.09)	0.58 (0.06)
	nSPCA RI	0.58 (0.08)	0.57 (0.09)	0.57 (0.07)

Diurnal changes improved from latanoprost baseline by adding dorzolamide in OA EDV by 1.4 ± 1.9, in OA RI by 0.03 ± 0.05 in younger and impaired by 0.39 ± 1.7 in OA EDV and by 0.02 ± 0.04 in OA RI in older glaucoma patients (*p* < 0.05; t test). By adding timolol OA RI decreased by 0.02 ± 0.05 in younger and increased by 0.02 ± 0.05 in older patients (*p* < 0.05). Patients younger than 65 years had statistically significant decreased morning OA RI from 0.79 ± 0.07 to 0.76 ± 0.08 in dorzolamide add-on therapy group (*p* < 0.05; t test). Patients older than 65 years had increased OA RI in the evening during both add-on therapies (0.77 ± 0.04 to 0.80 ± 0.05), (*p* < 0.05). Older patients group had bigger fluctuations in systolic BP, OPP, SPP and MAP with dorzolamide and timolol comparing to younger patients, but the difference when using dorzolamide was statistically significant (*p* < 0.05, Table 5).

**Table 5:** Systolic BP, MAP, SPP and OPP fluctuations in different age groups.

	**Systolic BP**		**MAP**		**SPP**		**OPP**	
	Timolol	Dorzola-mide	Timolol	Dorzola-mide	Timolol	Dorzola-mide	Timolol	Dorzola-mide
<65 year	14.5(7.3)	10.3(6.9)	8.52(5.3)	6.92(4.5)	13.8(6.6)	10.6(6.7)	6.09(3.3)	4.96(3.0)
≥65 year	17.0(10.6)	16.5(9.3)	12.8(6.8)	10.9(5.6)	17.2(9.8)	15.8(8.2)	7.28(4.4)	7.98(4.9)

BP–blood pressure; SPP–systolic perfusion pressure; MAP–mean arterial pressure; OPP–ocular perfusion pressure.

Diurnal latanoprost baseline RI parameters of OA, CRA, tSPCA, nSPCA were lower in patients receiving glaucoma treatment less than 5 years (N = 17), compared with patients receiving more than 5 years of glaucoma treatment (N = 18), but a statistically significant difference was seen only in CRA and tSPCA RI (*p* < 0.05). Patients with periods of illness of less than 5 years had lower fluctuations in OPP with dorzolamide add-on therapy, while patients with longer period of illness had lower fluctuations with timolol add-on therapy.

Patients using less than two systemic medications (N = 18) had better OA, CRA, tSPCA, nSPCA RI diurnal latanoprost baseline parameters than patients using more than two medications (N = 17), but the differences were not statistically significant (*p* < 0.05).

**Figure 2:**
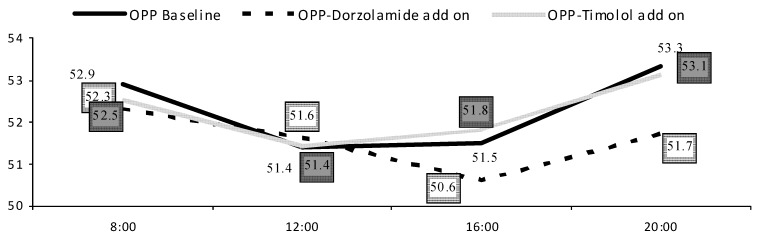
OPP diurnal fluctuations [mmHg].

**Figure 3:**
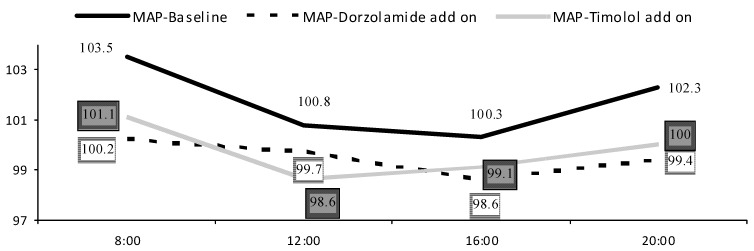
MAP diurnal fluctuations.

There were no statistically significant differences in mean BP parameters between treatment groups (*p* < 0.05). Most patients were on different systemic antihypertensive treatment receiving on average 2.3 (1.9) medications per day (Table 1). Dorzolamide add-on therapy showed smaller IOP (2.0 ± 1.4), SPP (13.3 ± 7.9), systolic BP (13.5 ± 8.7) and diastolic BP (8.4 ± 5.4) fluctuations as compared to both latanoprost baseline or timolol add-on therapies. Higher difference between morning and evening BP was correlated to decreased evening CRA EDV in timolol group (c = −0.41; *p* = 0.01). With increased MAP in the morning or evening hours, we found increased evening OA RI in timolol add-on group (c = 0.400; *p* = 0.02; c = 0.513; *p* = 0.002 accordingly). Higher MAP fluctuations were related to impaired RBF parameters during evening hours-decreased CRA EDV (c = −0.408; *p* = 0.01), increased CRA RI (c = 0.576; *p* < 0.001) and tSPCA RI (c = 0.356; *p* = 0.04) in the dorzolamide group and increased nSPCA RI (c = 0.351; *p* = 0.04) in the timolol add-on group. OPP fluctuations correlated with increased nSPCA RI (c = 0.453; *p* = 0.006) in the timolol group. OPP fluctuations were not related to IOP fluctuations in both add-on therapies (*p* < 0.05), but were related to BP fluctuations (Figures 2 and 3).

## 6. Discussion

Evaluating add-on effects of dorzolamide *vs*. timolol in patients already treated with latanoprost we found that both drugs statistically significantly lowered IOP, favouring timolol, but IOP fluctuations during the day decreased only with dorzolamide. According to AGIS patients with IOP < 14 mmHg had very little change in visual field defect score [14], and possibly at this level of IOP GON progression is stabilised. In our study we found that after adding-on IOP less than 14 mmHg was every 5th visit using timolol and every 7th visit using dorzolamide. 

As IOP therapies may influence ocular perfusion [15], it is vital to investigate glaucoma therapies for vascular interactions in addition to IOP reduction. In our study we found that during dorzolamide add-on therapy IOP, SPP, systolic BP and diastolic BP fluctuations were lower compared to both latanoprost baseline or timolol add-on therapies. In addition to vascular factors, IOP fluctuations are known to be increased in OAG [16]. Importantly, OPP is influenced by circadian IOP fluctuations [17]. Increased BP fluctuations may also induce ischemia if the autoregulation capacity of ocular tissue is exceeded. Furthermore, not the absolute BP level alone, but fluctuations of BP may account for deficits of OPP [16,17,18]. OPP fluctuations were related to BP, but not IOP fluctuations in both add-on therapies.This supports the idea that changes in IOP are not fully responsible for glaucomatous damage and variations in systemic BP are also a potential risk factor in glaucoma. In our study MAP fluctuations were linked to impaired retrobulbar blood flow parameters during evening hours in both add-on groups. Systemic BP plays an important role in maintaining the normal autoregulation of the optic nerve head and it may breakdown when systemic BP is altered [3].

According to our results patients with shorter illness periods had better response to dorzolamide, resulting in lower fluctuations of OPP, while patients with longer glaucoma treatment had the same effect with timolol. One might hypothesise that for a better treatment effect prescribing drugs according to glaucoma illness period should be taken into account.

In our study a statistically significant difference was found between younger and older patients in diurnal OA RI changes in both add-on groups. Younger patients using dorzolamide were linked to decreased morning OA RI and had lower fluctuations in OPP, SPP, MAP. This suggests better response to ocular hypotensive drugs in younger patients, probably due to a better vascular autoregulation. Our study findings may be explained by the vasodilatative effect of dorzolamide. Dorzolamide hydrochloride is perhaps the most vasoactive topical glaucoma treatment and several studies have shown it to increase various measures of ocular blood flow parameters [19,20,21,22,23,24], although other studies have failed to show similar effect [25,26,27]. Several authors when analysing normal subjects found that a single instillation of dorzolamide showed no significant effects on the blood flow through major retinal vessels [28,29,30,31,32]. There are also studies using CDI in subjects with POAG, OH or normal tension glaucoma that found no significant effects on the retrobulbar hemodynamics of a single instillation of timolol [33,34,35,36]. Stankiewicz *et al*. [37] found that adjunctive dorzolamide therapy to morning-dosed bimatoprost 0.03% reduced diurnal IOP and vascular resistance in the ophthalmic artery, but did not alter retinal circulation in patients with POAG. Siesky *et al*. showed that addition of dorzolamide to timolol monotherapy decreases IOP and increases retinal blood flow in the superficial retinal vasculature in both glaucomatous and healthy patients. The combination of increased retinal blood flow with consistent oxygen saturation may potentially increase oxygen delivery to the retina [38].

Our results showed that patients with shorter illness period or taking less systemic medications had lower baseline resistance indices, which results in better ocular blood flow and possibly indicates better glaucoma prognosis, even if some systemic drugs are thought to improve ocular circulation [39,40,41]. The occurrance of cardiovascular and cerebrovascular desease is the result of many pathological mechanisms, including endotelial dysfunction and autonomic nervous system disturbances. Therefore, glaucoma patients are often expected to also suffer from various systemic vascular pathologies. 

Possible limitations of the current study are a short observation period and specific limitations in the imaging technology used to assess OBF. Larger scale studies with longer follow up, standardization of OBF measurement techniques are required to elicit a clear understanding of vascular risk factors in glaucoma progression. 

## 7. Conclusions

Both dorzolamide and timolol add-on therapies statistically significantly lowered IOP but dorzolamide add-on therapy showed lower fluctuations in IOP, SPP and BP. Higher variability of daytime OPP led to impaired RBF parameters in the evening. With a variety of pharmacological agents the paradigm for today is to safely and most potentially treat individual glaucoma patient seeking for potential benefits in ocular perfusion.

## References

[1] Heijl A., Leske M.C., Bengtsson B., Bengtsson B., Hussein M., Early Manifest Glaucoma Trial Group (2002). Reduction of intraocular pressure and glaucoma progression (Results from the Early Manifest Glaucoma Trial). Arch. Ophthalmol..

[2] Leske C.M., Heijl A., Hyman L., Bengtsson B., Dong L., Yang Z., the EMGT Group (2007). Predictors of long-term progression in the early manifest glaucoma trial. Ophthalmology.

[3] Weinreb R.N., Harris A. (2009). Clinical Revalence of Ocular Blood Flow (OBF) Measurements Icluding Effects of General Medications of Specific Glaucoma Treatment. Ocular Blood Flow in Glaucoma.

[4] Klein B.E., Klein R., Knudtson M.D. (2005). Intraocular pressure and systemic blood pressure: Longitudinal prospective the Beaver Dam Eye Study. Br. J. Ophthalmol..

[5] Harris A., Jonescu-Cuypers C., Martin B., Kagemann L., Zalish M., Garzozi H.J. (2001). Simultaneous management of blood flow and IOP in glaucoma. Acta Ophthalmol. Scand..

[6] Plange N., Kaup M., Daneljan L., Predel H.G., Remky A., Arend O. (2006). 24-h Blood pressure monitoring in normal tension glaucoma: Night-time blood pressure variability. J. Hum. Hypertens..

[7] Liu J.H., Zhang X., Kripke D.F., Weinreb R.N. (2003). Twenty-four hour intraocular pressure pattern associated with early glaucomatous changes. Invest. Ophthalmol. Vis. Sci..

[8] Asrani S., Zeimer R., Wilesnsky J., Gieser D., Vitale S., Lindenmuth K. (2000). Large diurnal fluctuations in intraocular pressure are an independent risk factor in patients with glaucoma. J. Glaucoma.

[9] Quaranta L., Pizzolante T., Riva I., Haidich A.B., Konstas A.G., Stewart W.C. (2008). Twenty-four-hour intraocular pressure and blood pressure levels with bimatoprost *versus* latanoprost in patients with normal-tension glaucoma. Br. J. Ophthalmol..

[10] Elena P.P., Denis P., Kosina-Boix M., Saraux H., Lapalus P. (1990). Beta adrenergic binding sites in the human eye: An autoradiographic study. J. Ocul. Pharmacol..

[11] Cohen J.S., Khatana A.K., Greff L.J. (2004). Evolving paradigms in the medical treatment of glaucoma. Int. Ophthalmol..

[12] Moore D., Harris A., Wudunn D., Kheradiya N., Siesky B. (2008). Dysfunctional regulation of ocular blood flow: A risk factor for glaucoma?. Clin. Ophthalmol..

[13] Januleviciene I., Ehrlich R., Siesky B., Nedzelskiene I., Harris A. (2009). Visual function, optic nerve structure, and ocular blood flow parameters after 1 year of glaucoma treatment with fixed combinations. Eur. J. Ophthalmol..

[14] Agis Investigators (2000). The Advanced Glaucoma Intervention Study (AGIS): 7. The relationship between control of intraocular pressure and visual field deterioration. Am. J. Ophthalmol..

[15] Costa V.P., Harris A., Stefánsson E., Flammer J., Krieglstein G.K., Orzalesi N., Heijl A., Renard J.P., Serra L.M. (2003). The effects of antiglaucoma and systemic medications on ocular blood flow. Prog. Retin. Eye Res..

[16] Duke-Elder S. (1959). The phasic variation in the ocular tension in primary glaucoma. Am. J. Ophthalmol..

[17] Sacca S.C., Rolando M., Marletta A., Macrì A., Cerqueti P., Ciurlo G. (1998). Fluctuations of intraocular pressure during the day in open-angle glaucoma, normal-tension glaucoma and normal subjects. Ophthalmologica.

[18] Kaiser H.J., Flammer J., Graft T., Stumfig D. (1993). Systemic blood pressure in primary glaucoma. Graefes Arch. Clin. Exp. Ophthalmol..

[19] Siesky B., Harris A., Kagemann L., Stefansson E., McCranor L., Miller B., Bwatwa J., Regev G., Ehrlich R. (2010). Ocular blood flow and oxygen delivery to the retina in primary open-angle glaucoma patients: The addition of dorzolamide to timolol monotherapy. Acta Ophthalmol..

[20] Harris A., Arend O., Arend S., Martin B. (1996). Effects of topical dorzolamide on retinal and retrobulbar hemodynamics. Acta Ophthalmol. Scand..

[21] Martinez A., Gonzalez F., Capeans C., Perez R., Sanchez-Salorio M. (1999). Dorzolamide effect on ocular blood flow. Invest. Ophthalmol. Vis. Sci..

[22] Moss A.M., Harris A., Siesky B., Rusia D., Williamson K.M., Shoshani Y. (2010). Update and critical appraisal of combined timolol and carbonic anhydrase inhibitors and the effect on ocular blood flow in glaucoma patients. Clin. Ophthalmol..

[23] Siesky B., Harris A., Brizendine E., Marques C., Loh J., Mackey J., Overton J., Netland P. (2009). Literature review and meta-analysis of topical carbonic anhydrase inhibitors and ocular blood flow. Surv. Ophthalmol..

[24] Harris A., Jonescu-Cuypers C.P., Kagemann L., Nowacki E.A., Garzozi H., Cole C., Martin B. (2001). Effect of dorzolamide timolol combination *versus* timolol 0.5% on ocular blood flow in patients with primary open-angle glaucoma. Am. J. Ophthalmol..

[25] Bernd A.S., Pillunat L.E., Böhm A.G., Schmidt K.G., Richard G. (2001). Ocular hemodynamics and visual field in glaucoma treated with dorzolamide. Ophthalmologe.

[26] Pillunat L.E., Bohm A.G., Koller A.U., Schmidt K.G., Klemm M., Richard G. (1999). Effect of topical dorzolamide on optic nerve head blood flow. Graefes Arch. Clin. Exp. Ophthalmol..

[27] Bergstrand I.C., Heijl A., Harris A. (2002). Dorzolamide and ocular blood flow in previously untreated glaucoma patients: A controlled double-masked study. Acta Ophthalmol. Scand..

[28] Harris A., Evans D., Martin B., Zalish M., Kagemann L., McCranor L., Garzozi H. (2002). Nocturnal blood pressure reduction: Effect on retrobulbar hemodynamics in glaucoma. Graefes Arch. Clin. Exp. Ophthalmol..

[29] Galambos P., Vafiadis J., Vilchez S.E., Wagenfeld L., Matthiessen E.T., Richard G., Klemm M., Zeitz O. (2006). Compromised autoregulatory control of ocular hemodynamics in glaucoma patients after postural change. Ophthalmology.

[30] Grunwald J.E., Mathur S., DuPont J. (1997). Effects of dorzolamide hydrochloride 2% on the retinal circulation. Acta Ophthalmol. Scand..

[31] Faingold D., Hudson C., Flanagan J., Guan K., Rawji M., Buys Y.M., Trope G.E. (2004). Assessment of retinal hemodynamics with the Canon laser blood flowmeter after a single dose of 2% dorzolamide hydrochloride eyedrops. Can. J. Ophthalmol..

[32] Harris A., Spaeth G., Wilson R., Moster M., Sergott R., Martin B. (1997). Nocturnal ophthalmic arterial hemodynamics in primary open-angle glaucoma. J. Glaucoma.

[33] Nicolela M.T., Buckley A.R., Walman B.E., Drance S.M. (1996). A comparative study of the effects of timolol and latanoprost on blood flow velocity of the retrobulbar vessels. Am. J. Ophthalmol..

[34] Evans D.W., Harris A., Cantor L.B. (1999). Primary open-angle glaucoma patients characterized by ocular vasospasm demonstrate a different ocular vascular response to timolol *versus* betaxolol. J. Ocul. Pharmacol. Ther..

[35] Galassi F., Sodi A., Renieri G., Ucci F., Pieri B., Harris A., Siesky B. (2002). Effects of timolol and dorzolamide on retrobulbar hemodynamics in patients with newly diagnosed primary open-angle glaucoma. Ophthalmologica.

[36] Harris A., Spaeth G.L., Sergott R.C., Katz L.J., Cantor L.B., Martin B.J. (1995). Retrobulbar arterial hemodynamic effects of betaxolol and timolol in normal-tension glaucoma. Am. J. Ophthalmol..

[37] Stankiewicz A., Misiuk-Hojło M., Grabska-Liberek I., Romanowska-Dixon B., Wierzbowska J., Mulak M., Szuścik I., Sierdziński J., Ehrlich R., Harris A. (2011). Intraocular pressure and ocular hemodynamics in patients with primary open-angle glaucoma treated with the combination of morning dosing of bimatoprost and dorzolamide hydrochloride. Acta Ophthalmol..

[38] Siesky B., Harris A., Kagemann L., Stefansson E., McCranor L., Miller B., Bwatwa J., Regev G., Ehrlich R. (2010). Ocular blood flow and oxygen delivery to the retina in primary open-angle glaucoma patients: The addition of dorzolamide to timolol monotherapy. Acta Ophthalmol..

[39] Steigerwalt R.D., Belcaro G.V., Laurora G., Cesarone M.R., de Sanctis M.T., Incandela L. (1998). Ocular and orbital blood flow in patients with essential hypertension treated with trandolapril. Retina.

[40] Spicher T., Orgul S., Gugleta K., Teuchner B., Flammer J. (2002). The effect of losartan potassium on choroidal haemodynamics in healthy subjects. J. Glaucoma.

[41] Resch H., Weigert G., Karl K., Pemp B., Garhofer G., Schmetterer L. (2009). Effect of systemic moxaverine on ocular blood flow in humans. Acta Ophthalmol..

